# Bone restoration after revision hip arthroplasty with femoral bone defects using extensively porous-coated stems with cortical strut allografts

**DOI:** 10.1186/s13018-020-01720-8

**Published:** 2020-05-27

**Authors:** Zichuan Ding, Tingxian Ling, Ping Mou, Duan Wang, Kai Zhou, Zongke Zhou

**Affiliations:** Department of Orthopedics, West China Hospital/West China School of Medicine, Sichuan University, 37# Wuhou Guoxue Road, Chengdu, People’s Republic of China

**Keywords:** Revision THA, Bone remodeling, Femoral bone defects, Extensively porous-coated stems, Cortical strut allografts

## Abstract

**Background:**

Stress shielding and bone loss of the femur are of great concern after revision total hip arthroplasty (THA) with extensively porous-coated stems, especially in a femur with already bone loss. The femoral bone remodeling patterns after revision THA with femoral bone defects using extensively porous-coated stems with cortical strut allografts remain unclear.

**Methods:**

We retrospectively reviewed 47 patients who underwent revision THA using extensively porous-coated stems combined with cortical strut allografts and 75 patients without allografts. The minimum follow-up was 2 years. Femoral bone remodeling signs, including stress shielding, bone restoration in bone defect area, distal cortical hypertrophy, and femoral width, were compared between patients with and without cortical strut allografts. Clinical outcomes were also compared between two groups.

**Results:**

Patients with cortical strut allografts showed less severe stress shielding (*P* = 0.01) than patients without allografts. Patients with allografts had more osseous restoration in bone defect area than patients without allografts (63.8% vs 30.7%, *P* < 0.001). Femoral width was significantly higher in femur with allografts than in femur without allografts at the immediate postoperative stage and latest follow-up (both *P* < 0.001). The hip function score, re-revision rate, and complications were comparable between two groups.

**Conclusion:**

The application of cortical strut allografts can decrease the severity of stress shielding, augment osseous restoration in bone defect area and improve femoral bone stock after revision THA using extensively porous-coated stems.

## Introduction

Aseptic loosening, infection, osteolysis, periprosthetic fracture, stress shielding, and implant removal can result in femoral bone defects that must be addressed at time of revision total hip arthroplasty (THA). Extensively porous-coated stems have been the predominant choice for revision THA with femoral bone defects. They can bypass the proximal bone defect region and achieve scratch fit fixation in the diaphysis and have shown reliable clinical outcomes in revision THA with bone loss [1–5]. However, stress shielding and further bone loss of the femur are of great concern after revision THA with extensively porous-coated stems, especially in femur with already extensive bone defects. The reported rates of severe stress shielding after revision THA using extensively porous-coated stems ranged from 6 to 22% [1–6]. The cementless long extensively porous-coated stem with high stiffness distributes some of the loads to the distal femur, and lack of stress in the proximal metaphyseal region leads to bone loss. As a result, proximal osteopenia of stress shielding and distal cortical hypertrophy of stress transfer was often observed. Besides, whether osseous restoration in bone defect area and improvement in femoral bone stock after revision THA using extensively porous-coated stems can be achieved is uncertain [6, 7]. Poor femoral bone stock after revision THA influences the functional outcomes [8, 9], increases the risk of aseptic loosening [10], increases the risk of periprosthetic femoral fracture (PFF) [11], and presents particular problems if further revision is required [12]. Improvement in femoral bone stock after revision surgery is of vital importance to eliminate the correlation between severe bone defects and poor clinical outcomes [13].

Cortical strut allografts can successfully reconstruct the bone stock in revision THA. Satisfactory clinical outcomes of revision THA using extensively porous-coated stems combined with cortical strut allografts have been reported [14–17]. It has been demonstrated that incorporated cortical strut allografts can undergo revascularization, be replaced with host bone, regain considerable strength, and undergo adaptive bone remodeling [13, 18–21]. Lack of stress loading on the cortical strut allografts can lead to bone loss of allografts, suggesting cortical strut allografts can also develop stress shielding [15, 22, 23]. However, whether additional application of cortical strut allografts can decrease the severity of stress shielding and promote bone restoration when compared to using extensively porous-coated stems alone remains unclear.

The purpose of this study was to investigate the femoral bone remodeling patterns, including stress shielding, bone restoration in bone defect area, distal cortical hypertrophy, and femoral width, after using extensively porous-coated stems with cortical strut allografts by comparing with those using extensively porous-coated stems alone. Clinical outcomes were also investigated in patients with and without cortical strut allografts.

## Patients and methods

We retrospectively reviewed 144 patients who underwent revision THA at our institution from January 2010 to July 2017. A total of 15 patients were lost to follow-up after surgery, and five patients had died, with no deaths related to the revision THA. Two patients were reached by telephone at last follow-up and were all satisfied with their hip function but refused to return for follow-up. The remaining 122 patients with a minimum 2-year follow-up were enrolled in this study. All patients were diagnosed with Paprosky type II, III, and IV femoral bone defects. Extensively porous-coated stems combined with cortical strut allografts were applied in 47 patients (allografts group), and stems alone in 75 patients (no allografts group). No significant difference in demographic data was observed between two groups (Table 1). The institutional review board of our hospital approved this retrospective study. All patients provided informed consent for participation.

**Table 1:** Demographic data

Parameters	Allografts group (*n* = 47)	No allografts group (*n* = 75)	*P* value
Male	29 (61.7%)	44 (58.7%)	0.739
Age	57.2 ± 16.5 (29–83)	53.5 ± 17.4 (33–87)	0.196
Follow-up	6.4 ± 2.5 (2.3–11.5)	6.0 ± 2.2 (2.2–11.8)	0.302
Primary diagnosis			0.958
Osteonecrosis of the femoral head	25 (53.2%)	39 (52.0%)	
Developmental dysplasia of the hip	12 (25.5%)	16 (21.3%)	
Primary osteoarthritis	6 (12.8%)	11 (14.7%)	
Femoral neck fractures	3 (6.4%)	7 (9.3%)	
Rheumatoid arthritis	1 (2.1%)	2 (2.7%)	
Mean time from primary to revision THA	9.3 ± 4.2 (1–18)	8.9 ± 4.9 (0–17)	0.824
Reason for revision			0.575
AL	14 (29.8%)	29 (38.7%)	
PJI	16 (34.0%)	24 (32.0%)	
PFF	17 (36.2%)	22 (29.3%)	
Fixation of previous femoral stems			0.739
Cement	29 (61.7%)	44 (58.7%)	
Cementless	18 (38.3%)	31 (41.3%)	
Degree of femoral bone defects (Paprosky classification)			0.106
Type II	9 (19.1%)	20 (26.7%)	
Type IIIA	21 (44.7%)	36 (48.0%)	
Type IIIB	12 (25.5%)	19 (25.3%)	
Type IV	5 (10.6%)	0 (0%)	
ETO utilized	10 (21.3%)	14 (18.7%)	0.724

Categorical variables are presented as numbers (percentage). Continuous variables are presented as the means ± standard deviations (range). *AL* aseptic loosening, *PJI* periprosthetic joint infection, *PFF* periprosthetic femoral fracture, *ETO* extended trochanteric osteotomy

All femoral components were revised with cementless extensively porous-coated solution stems (DePuy, Warsaw Indiana). When traditional methods fail in attempting to extract a well-fixed stem, extended trochanteric osteotomy (ETO) was performed. When the initial axial and rotational stability of the stems could not be achieved, or the area of cortical bone defects was large, or the thin cortical bone presented a high risk of intraoperative fracture, cortical strut allografts were used. One to three cortical strut allografts were used in different sides of the femur, usually the lateral, medial, or anterior femur, which can be observed in Gruen zone 1-11. The allografts used in these operations were made from tibial bone and previously stored at − 80 °C for at least 3 months in the bone bank of our institution. The allografts were soaked in povidone-iodine solution repeatedly and finally coated with dry-powdered gentamicin and vancomycin, which was all performed on another surgical table under sterile condition. Except use of cortical strut allografts, other techniques and therapy protocol were the same in both groups [24]. Generally, patients were mobilized with partial weight-bearing 1 to 4 weeks after the operation, depending on the degree of preoperative bone defects. Full weight-bearing and ambulation without crutches were allowed after 4 to 12 weeks. Patients were followed up clinically and radiographically after surgery at regular intervals of 1, 2, 3, 6, and 12 months as well as annually thereafter.

Anteroposterior and lateral radiographs of the femurs at each follow-up time point along with preoperative radiographs were taken and reviewed. Areas around the femoral stem were divided into 14 zones as described by Gruen et al. [25]. The area of stress shielding was assessed according to the criteria of Engh and Bobyn [26] and partially modified by Kusano et al. [8]. A small area of stress shielding was defined as bone loss in only Gruen zones 1, 7, 8, or 14; a moderate area of stress shielding as any bone loss in Gruen zones 2, 6, 9, or 13; and a large area of stress shielding as bone loss in the remaining Gruen zones: 3, 4, 5, 10, 11, or 12. In addition, we assessed the severity of stress shielding based on bone density according to Moreland and Moreno [27]. Mild stress shielding was indicated when little change in cortical density and thickness was observed. Moderate stress shielding was indicated by significant and obvious loss of cortical density and thickness, and severe stress shielding was indicated by a major, striking, and impressive degree of bone loss. When the cortex of the host bone was covered by allografts, stress shielding was evaluated by comparing earliest radiographs showing that cortical strut allografts were united to the host bone with latest radiographs. Bone restoration in bone defect area was classified as osseous restoration, constant defects, or increasing defects, according to the criteria of Bohm and Bischel [28]. Distal cortical hypertrophy was assessed as previously described by Ritter and Fechtman [29]. Femoral width was measured at the zone with the most severe bone loss, where cortical struts allografts were always applied to augment the bone stock in the allografts group [30].

The fixation and stability of the cementless femoral components were evaluated according to the criteria of Engh et al. [31]. Incorporation of cortical strut allografts to host bone was defined as complete union and bridging between them. Fracture nonunion was defined as a persistent fracture line or absence of bridging callus after six postoperative months [32]. Hip function evaluation was conducted by two observers not involved with the surgical and clinical care of the patients using Harris Hip Score (HHS). Any re-revisions and intraoperative and postoperative complications were recorded.

Two independent observers quantified radiographic parameters to compare the interobserver reliability. Intraobserver reliability was assessed by repeated measurement by the first author on separate occasions, 2 weeks apart. Reliability analysis showed high interobserver and intraobserver agreements for all radiographic parameters, with intraclass correlation coefficient (ICC) over 0.75. Student’s *t* test was utilized to analyze continuous variables. Mann-Whitney *U* tests and Pearson chi-square tests were performed to analyze ordinal and unordered categorical variables, respectively. The correlation between two ordinal categorical variables was analyzed by Spearman’s rank correlation coefficient test. The level of significance was defined as *P* < 0.05. Statistical analysis was performed with SPSS v22.0 (IBM, Armonk, NY).

## Results

**Fig. 1:**
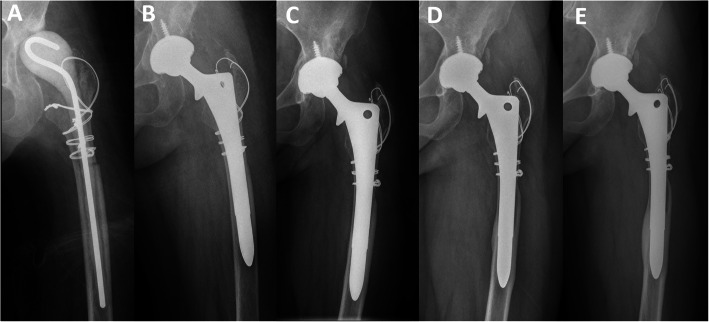
Anteroposterior radiographs of a 48-year-old woman who underwent revision THA for periprosthetic joint infection. **a** Radiograph prior to stage 2 revision THA showing the antibiotic-loaded spacer in situ. **b** Radiograph immediately after revision THA using a cementless extensively porous-coated stem without cortical strut allografts. **c** Postoperative radiograph at 1 year showing loss of cortical density and thickness in the proximal femur, suggesting moderate area and severity of stress shielding. **d** Postoperative radiograph at 5 years showing severe stress shielding and distal cortical hypertrophy on both the medial and lateral sides. **e** Postoperative radiograph at 10 years showing more severe bone loss and distal cortical hypertrophy

**Fig. 2:**
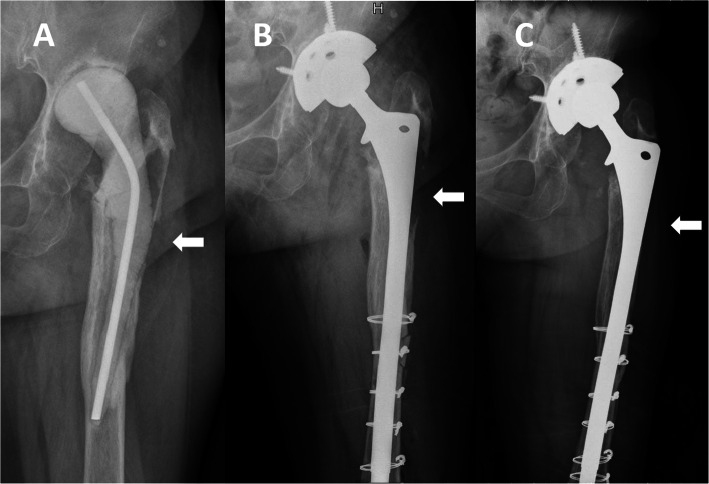
Anteroposterior radiographs of a 76-year-old man who underwent revision THA for a periprosthetic joint infection using extensively porous-coated stem alone. **a** Radiograph prior to stage 2 revision THA showing a Paprosky type IIIB femoral bone defect. **b** Postoperative radiograph at 6 months showing no bone restoration in the bone defect area (white arrow). **c** Postoperative radiograph at 5 years showing bone defects still existing and moderate stress shielding on both the medial and lateral sides

**Fig. 3:**
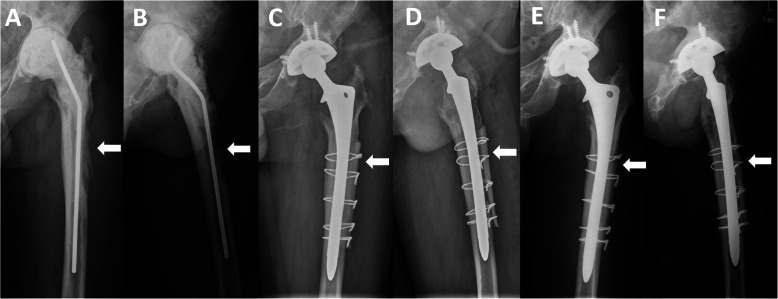
Radiographs of a 44-year-old man who underwent revision THA for periprosthetic joint infection. **a**, **b** Anteroposterior and lateral radiographs prior to stage 2 revision THA showing a Paprosky type IIIA femoral bone defect. **c**, **d** Radiographs immediately after revision THA using an extensively porous-coated stem with cortical strut allografts, showing that the cortical strut allografts bridged the bone defects (white arrow) and supported the thinning cortex. **e**, **f** Postoperative radiographs at 9 years showing successful incorporation of the cortical strut allografts to the host bone, bone restoration in the bone defect area (white arrow), and a significant increase in femoral width. No sign of stress shielding and distal cortical hypertrophy was observed. The stem was assessed as bone ingrowth stable

Patients with cortical strut allografts showed less severe stress shielding than patients without allografts (*P* = 0.01) (Table 2, Figs. 1, 2, and 3). The area of stress shielding was smaller in the allografts group than in the no allografts group, but it did not reach statistical significance (*P* = 0.599). The severity of stress shielding was positively correlated with preoperative Paprosky classification of femoral bone defects (Spearman’s rank correlation coefficient = 0.796, *P* < 0.001), while the area of stress shielding was not associated with preoperative bone defects (Spearman’s rank correlation coefficient = 0.14, *P* = 0.326). No difference was observed in HHS among different severities of stress shielding (analysis of variance [ANOVA], *P* = 0.424) and different areas of stress shielding (ANOVA, *P* = 0.328).

**Table 2:** Outcome parameters

Parameters	Allografts group (*n* = 47)	No allografts group (*n* = 75)	*P* value
Area of stress shielding			0.599
Small	28 (59.5%)	41 (54.7%)	
Moderate	13 (27.7%)	23 (30.7%)	
Large	6 (12.8%)	11 (14.7%)	
Severity of stress shielding			0.01
Mild	40 (85.1%)	48 (64.0%)	
Moderate	5 (10.6%)	16 (21.3%)	
Severe	2 (4.3%)	11 (14.7%)	
Distal cortical hypertrophy	14 (29.8%)	29 (38.7%)	0.318
Bone restoration in the defect area			< 0.001
Osseous restoration	30 (63.8%)	23 (30.7%)	
Constant defects	12 (25.5%)	30 (40.0%)	
Increasing defects	5 (10.6%)	22 (29.3%)	
Femoral width (mm)*			
Pre-operation	31.7 ± 4.4 (22.6–38.0)	32.2 ± 5.1 (23.9–39.4)	0.457
Immediate post-operation	42.1 ± 6.5 (29.9–55.6)	32.3 ± 5.0 (23.8–39.4)	< 0.001
Latest follow-up	38.6 ± 7.2 (25.3–57.7)	31.2 ± 6.5 (22.3–42.5)	< 0.001
Fixation and stability of the stems			0.672
Stable bone ingrowth	40 (85.1%)	59 (78.7%)	
Stable fibrous ingrowth	6 (12.8%)	14 (18.7%)	
Unstable	1 (2.1%)	2 (2.7%)	
Re-revision	2 (4.3%)	3 (4.0%)	0.945
PJI	0 (0%)	1 (1.3%)	
AL	1 (2.1%)	2 (2.7%)	
PFF	1 (2.1%)	0 (0%)	
HHS			
Preoperative points	41.2 ± 10.7	43.7 ± 9.9	0.281
Postoperative points	84.6 ± 6.0	84.8 ± 6.6	0.495
Complications	4 (8.5%)	5 (6.7%)	0.705
Intraoperative fracture	2 (4.3%)	3 (4.0%)	
Wound infection	1 (2.1%)	0 (0%)	
Postoperative dislocation	1 (2.1%)	2 (2.7%)	

*P value was analyzed by the paired *t* test

Patients with cortical strut allografts had more osseous restoration in bone defect area than patients without allografts (63.8% vs 30.7%, *P* < 0.001) (Table 2, Figs. 2 and 3). Osseous restoration was positively associated with ETO (Pearson chi-square test, *P* = 0.014). The occurrence of distal cortical hypertrophy showed no difference between the allografts group and no allografts group (29.8% and 38.7%, respectively) (Fig. 1). Femoral width was significantly higher in femurs with allografts than in femurs without allografts at the immediate postoperative stage and latest follow-up (both *P* < 0.001). Compared to the preoperative width, the femoral width at the latest follow-up increased by a mean of 6.9 mm (21.8% of preoperative width) in the allografts group, while the femoral width decreased by 1.1 mm in the no allografts group at the latest follow-up.

No difference was observed concerning signs of instability in both groups (Table 2). Incorporation of cortical strut allografts to host bone was confirmed in all 47 patients at a mean follow-up of 9.1 months. All PFFs and ETOs achieved union. The re-revision rates were comparable between the allografts and no allografts groups (4.3% vs 4.0%, *P* = 0.945). One PFF occurred in the allografts group and one acute hematogenous periprosthetic joint infection occurred in the no allografts group, but both stems were evaluated as stable in re-revision surgery. The HHS showed significant improvement after revision THA in both groups (both *P* < 0.001). No significant difference was found in intraoperative and postoperative complications between two groups.

## Discussion

Stress shielding and further bone loss of the femur with already deficient bone stock are of great concern after revision THA using extensively porous-coated stems [1–5]. Enhancement of femoral bone stock after revision THA can improve functional outcomes, decrease the risk of PFF and aseptic loosening, and render further re-revision easier [8–13]. Cortical strut allografts were reported to successfully reconstruct the femur and improve bone stock in revision THA with bone defects [14–16]. However, whether additional application of cortical strut allografts can decrease the severity of stress shielding and promote bone restoration when compared to using extensively porous-coated stems alone remains unclear. To the best of our knowledge, no previous studies have compared the femoral bone remodeling patterns after revision THA using extensively porous-coated stems with and without cortical strut allografts.

Although we found that extensively porous-coated stems with and without cortical strut allografts can both provide satisfactory clinical outcomes after revision THA with bone defects, the most striking finding in our study was that additional application of cortical strut allografts can further decrease the severity of stress shielding, augment osseous restoration in bone defect area, and reconstruct femoral bone stock. Although we did not find any difference in functional outcomes among patients with different degrees of stress shielding, we speculated that severe bone loss may result in worse hip function at longer follow-up time, as previously described [8, 9]. Decreasing the severity of stress shielding and improving bone stock can eliminate the correlation between severe bone defects and poor clinical outcomes [13]. Besides, we believe that the promotion of bone restoration after application of allografts can provide superior longevity of revision stem and decrease the risk of aseptic loosening and periprosthetic fracture at longer follow-up time [10–12]. Moreover, application of allografts can provide a better bone stock situation when a further revision is required. Since we found the severity of stress shielding was positively correlated with degree of preoperative bone defects, which is in accordance with previous findings [1, 2, 33], we suggest, in revision THA cases with severe femoral bone defects, use of cortical strut allografts is a reliable way to reduce bone loss, promote bone restoration and improve femoral bone stock.

Many previous studies have assessed the severity of stress shielding around extensively porous-coated stems based on the area of stress shielding. The reported rates of severe stress shielding, defined as a large area of bone loss extending to the femoral diaphysis, ranged from 6 to 22% [1–6]. In this study, stress shielding extending to the femoral diaphysis was observed in 12.8% and 14.7% of patients in the allografts group and no allografts group, respectively. However, it did not reach statistical significance. We also investigated the severity of stress shielding based on the intensity of bone loss and found patients with cortical strut allografts had a significantly lower rate of severe stress shielding than patients without allografts (4.3% vs 14.7%; P = 0.01). In summary, the application of allografts decreased the severity of stress shielding and delayed the process of stress shielding, although stress shielding still developed at the united allografts and host bone.

The decreased severity and delayed process of stress shielding after application of cortical strut allografts may be explained as follows. First, the high rate of incorporation of cortical strut allografts leads to significant increase of bone mass, which can limit the influence of stress shielding on bone stock. Second, it has been demonstrated that after union occurs, cortical strut allografts can be invaded by osteoclasts, osteoblasts, and vascular and be replaced with host bone and maintain biologically active, thus further responding to stress and undergoing adaptive bone remodeling [13, 18] Cortical strut allografts change the biological environment of host bone and coordinate the host bone to respond to stress. Third, the stress transfer in stem and host bone depends on the stiffness of the stem and host bone [34]. Use of cortical strut allografts leads to an increase in the stiffness of host bone and consequently enhances the stress transfer in host bone [35, 36]. The cortical strut allografts have considerable strength and similar modulus of elasticity as host bone, thus minimizing modulus mismatch and decrease stress shielding in the host bone [20, 37].

Although bone restoration in bone defect area is expected after revision using cementless stems alone, Richards et al. [7] and Kimura et al. [38] reported that the osseous restoration in bone defect area only occurred in 8.1% and 50.0% of the patients, respectively. This study has shown 63.8% of the femurs in the allografts group and 30.7% of the femurs in the no allografts group had osseous restoration. Successful incorporation of allografts to host bone leads to augmentation of osseous restoration by a large margin. Osseous restoration was positively associated with ETO, which can be explained by the fracture healing process caused by ETO, including cytokine secretion and vascular invasion [32, 33]. In the allografts group, we found a significant enhancement of femoral width, which reflected the reconstruction of the femoral bone stock through the application of cortical strut allografts. When comparing the immediate postoperative and latest follow-up femoral widths to the preoperative femoral width, an increase of 10.4 mm and 6.9 mm, respectively, was observed. This result was in agreement with the previously reported data [30, 39]. A slight decrease in the femoral width (3.5 mm) was observed after 6-year follow-up, which was consistent with the stress shielding developed in allografts and host bone. In contrast to patients in the allografts group, patients in the no allografts group showed decreased femoral width over time.

Distal cortical hypertrophy, seen with new cortical bone formation and increase in distal cortical thickness, suggests a transfer of the majority of the load from the distal part of stem to the femur [29]. The distal cortical hypertrophy occurred less in the allografts group than in the no allografts group in our study (29.8% vs 38.7%), suggesting that use of cortical strut allografts led to an enhancement of loading transfer in proximal host bone and consequently decreased the load transferred from the distal part of stem to the femur. However, it did not reach statistical significance (*P* = 0.318). Distal cortical hypertrophy and stress shielding are similar phenomena which are caused by the modified femoral loading patterns after revision THA using extensively porous-coated stems. Cortical strut allografts seem to be a reliable approach to change the mechanical and biological environment of host bone and improve the bone remodeling patterns.

The present study has several limitations. First, a mean follow-up time of 6.1 years was relatively short. It might be inadequate to determine the influence of radiographic changes on clinical outcomes and distinguish the difference between two groups. Second, the minimum follow-up time was only 2 years. However, we found the bone remodeling progressed little with time over 2 years, which was in accordance with previously reported by Engh and Bobyn [26]. Third, the bone remodeling patterns were assessed on plain radiographs in our study. Dual-energy X-ray absorptiometry or computed tomography can detect and quantify bone density changes with high precision and reproducibility. Further studies with longer follow-up and more precise detection methods are required to investigate the femoral bone remodeling patterns after using cortical strut allografts.

## Conclusion

In conclusion, the application of cortical strut allografts can decrease the severity of stress shielding, augment osseous restoration in bone defect area, and improve femoral bone stock after revision THA using extensively porous-coated stems. We suggest, in revision THA cases with severe femoral bone defects, use of cortical strut allografts is a reliable way to reduce bone loss, promote bone restoration, and improve femoral bone stock.

## Data Availability

The datasets analyzed during the current study are available from the corresponding author on reasonable request.

## Abbreviation

THA: Total hip arthroplasty
ETO: Extended trochanteric osteotomy
PFF: Periprosthetic femoral fracture
HHS: Harris Hip Score
ICC: Intraclass correlation coefficient
ANOVA: Analysis of variance
